# Targeting protein O‐Glcnacylation to rescue cognitive defects and Alzheimer‐like signatures in a mouse model of down syndrome

**DOI:** 10.1002/alz.086816

**Published:** 2025-01-03

**Authors:** Fabio Di Domenico, Chiara Lanzillotta, Francesca Prestia, Antonella Tramutola, Eugenio Barone, Marzia Perluigi

**Affiliations:** ^1^ Sapienza University of Rome, Rome, Rome Italy

## Abstract

**Background:**

Disturbances of protein O‐GlcNAcylation have pointed out as a possible link between altered brain metabolism and cognitive decline. We previously demonstrated the disruption of O‐GlcNAcylation homeostasis, as an effect of altered OGT and OGA regulatory mechanism, and we confirmed the relevance of O‐GlcNAcylation in the appearance of Alzheimer disease hallmarks in the brain of a murine model of Down syndrome (DS). Furthermore, we provide evidence for the neuroprotective effects of brain‐targeted OGA inhibition (Thiamet G). The primary objective of this study is to provide evidence for the neuroprotective effects of brain‐targeted OGA inhibition (Thiamet G) by analyzing mice cognition and molecular pathways associated with Alzheimer‐like neurodegenenration.

**Method:**

The neuroprotective effects of Thiamet G was evaluated in DS mice by analyzing mice performances in the novel object recognition and Y maze tests. Furthermore we analyzed by immunochemical methods APP and tau modification, and by proteomic approach using ESI‐MS/MS technique**,** we identified brain proteins whose O‐GlcNAcylation levels resulted significantly modulated by the treatment.

**Result:**

Data supported the beneficial effect Thiamet G in DS mice hippocampus. The rescue of OGA activity was able to restore protein O‐GlcNAcylation, rescue cognitive impairments and reduce AD‐related hallmarks. In particular, the recovery of O‐GlcNAcylation was associated with the modulation of protein specific O‐GlcNAc levels occurring to several components of neuronal architecture, stress response mechanisms and energy production.

**Conclusion:**

Our work emphasizes the central role of altered protein O‐GlcNAcylation in DS neuropathology and lays the foundations to consider the rescue of protein O‐GlcNAcylation as a valuable therapeutic strategy to reduce the alterations of brain metabolism and the development of AD‐hallmarks.

